# Institutional Notes and News

**Published:** 1911-07-29

**Authors:** 


### INSTITUTIONAL NOTES AND NEWS

**New Dental School for the London.**

At the distribution of prizes to the students of the London Hospital Medical College, in the presence of the American Ambassador, last week, who was welcomed by Mr. W. Douro Hoare, Chairman of the College Ward, the Dean (Dr. W. Wright) drew attention to the opening next October of a dental school in connection with the College. It will then be claimed that everything, except infective fevers, can be studied at the London. A resident hostel for some of the students has been opened also. Dr. Wright also anticipated that the grant of £800 received from the Treasury would be largely increased. The Ambassador stated the Bellevue Hospital in New York was larger than the London, when Mr. Holland reminded the audience that the Bellevue, unlike the London, was supported by the State.

**Decorations for the Royal Waterloo Staffs.**

Sir E. Durning-Lawrence, Chairman of the Royal Waterloo Hospital for Children and Women, Mr. Topham Richardson, Hon. Treasurer, and Mr. H. H. Franklyn, Secretary, received the Duchess of Albany last week on the occasion of the Coronation fête organised for the patients and the staff. Her Royal Highness made a tour of the wards, and presented Coronation badges to the medical and nursing staffs in Ward III., and to the domestic staff in her own, the Duchess of Albany Ward. Afterwards she attended the dedication service at the opening of the Isolation Ward, which was conducted by the Rev. E. G. Gordon, chaplain to the hospital. The Duchy of Cornwall owns this building, which is one of its earliest improvements to its Lambeth property, and which has replaced a public-house. The ladies' association was represented by Mrs. E. D. Harkness, its Hon. Secretary.

